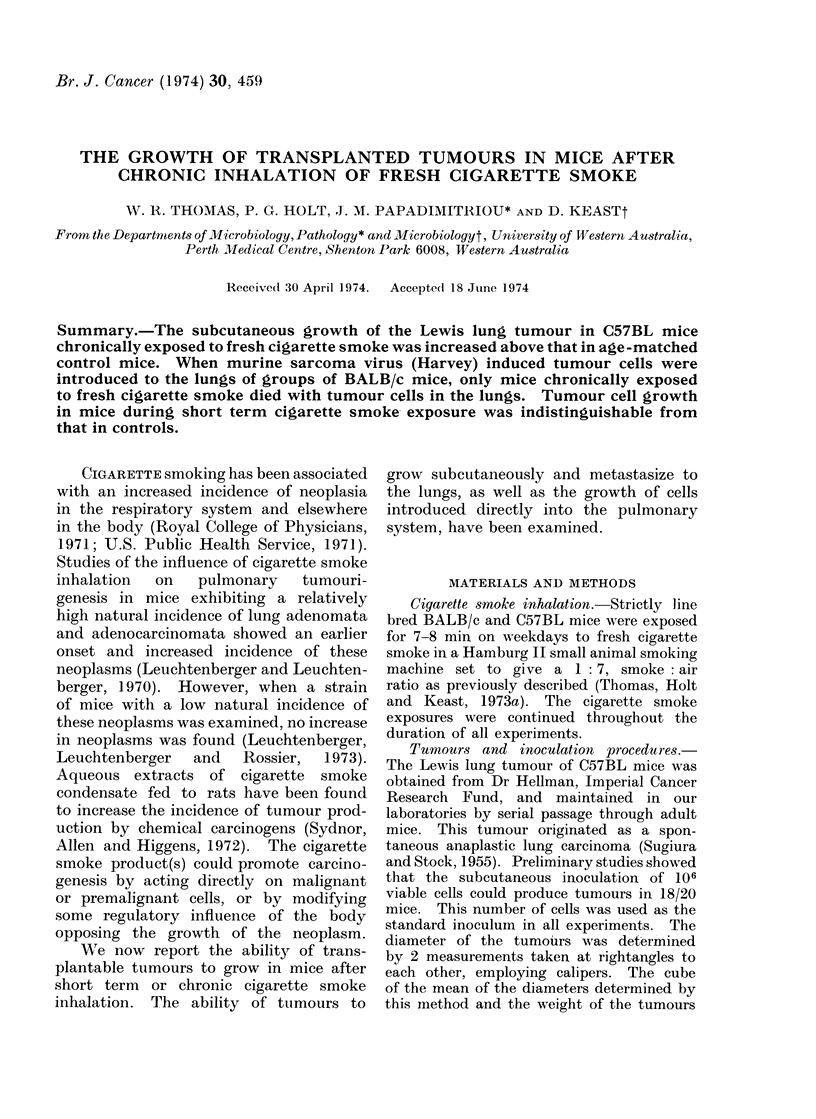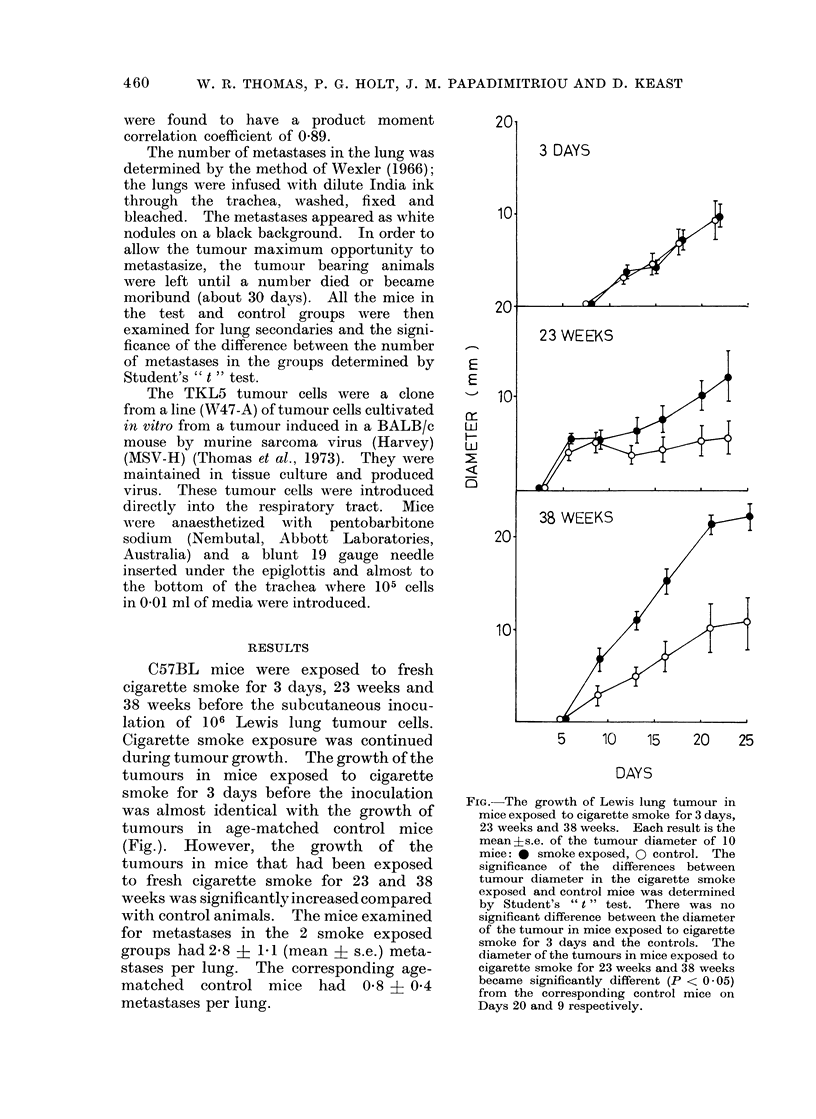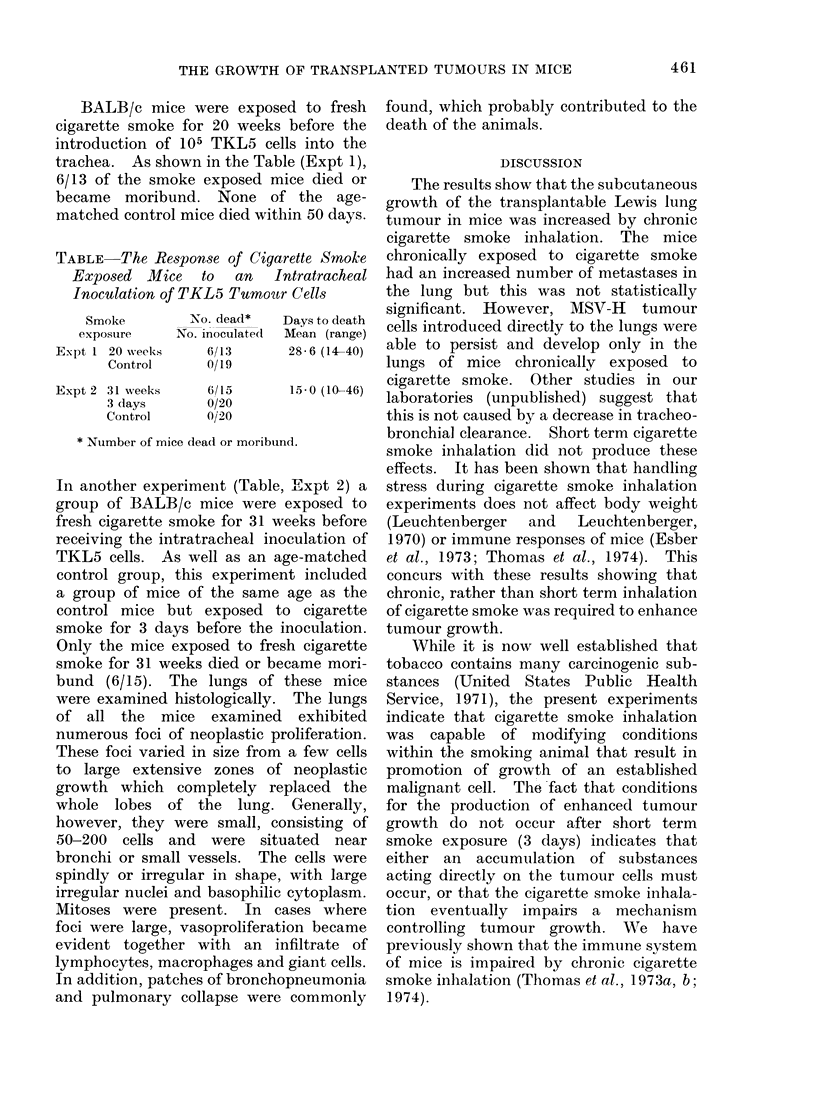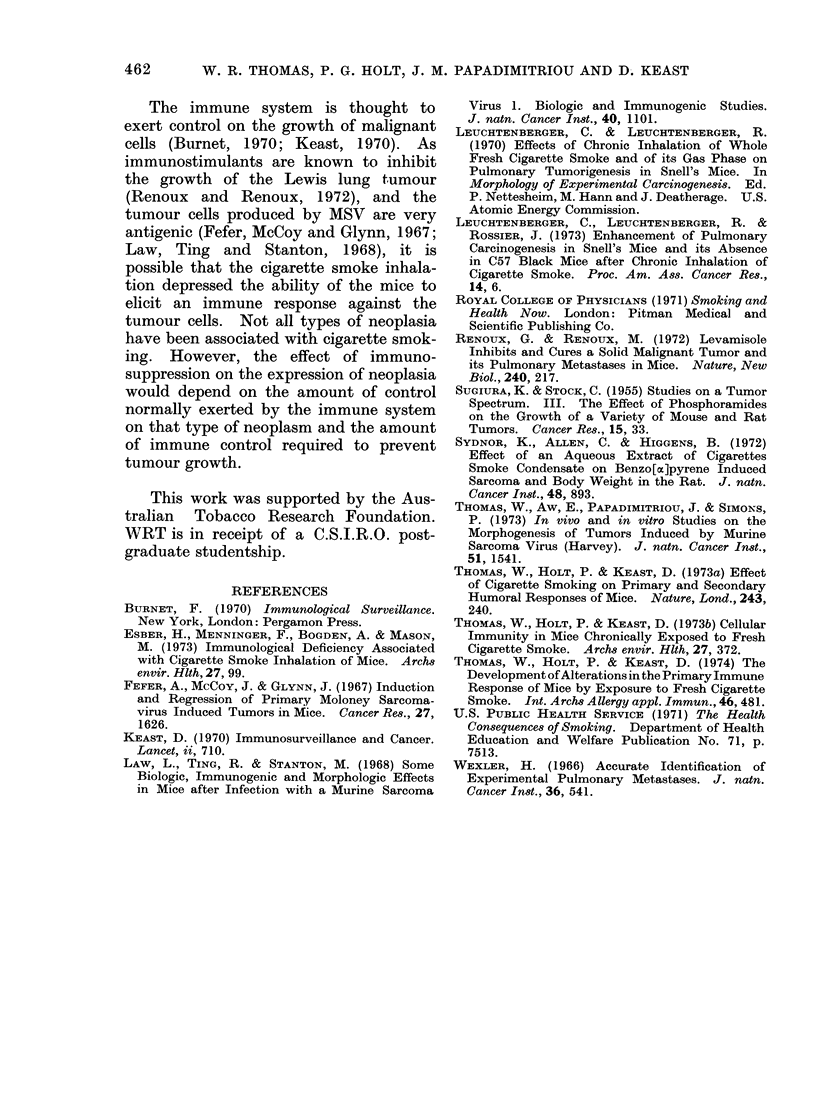# The Growth of Transplanted Tumours in Mice after Chronic Inhalation of Fresh Cigarette Smoke

**DOI:** 10.1038/bjc.1974.220

**Published:** 1974-11

**Authors:** W. R. Thomas, P. G. Holt, J. M. Papadimitriou, D. Keast

## Abstract

The subcutaneous growth of the Lewis lung tumour in C57BL mice chronically exposed to fresh cigarette smoke was increased above that in age-matched control mice. When murine sarcoma virus (Harvey) induced tumour cells were introduced to the lungs of groups of BALB/c mice, only mice chronically exposed to fresh cigarette smoke died with tumour cells in the lungs. Tumour cell growth in mice during short term cigarette smoke exposure was indistinguishable from that in controls.


*Br. J. Cancer* (1974) **30**, 459

### THE GROWTH OF TRANSPLANTED TUMOURS IN MICE AFTER CHRONIC INHALATION OF FRESH CIGARETTE SMOKE

W. R. THOMAS, P. G. HOLT, J. M. PAPADIMITRIOU* AND D. KEAST†

*From the Departments of Microbiology, Pathology* and Microbiology†, University of Western Australia, Perth Medical Centre, Shenton Park* 6008, *Western Australia*

Received 30 April 1974. Accepted 18 June 1974

**Summary.—The subcutaneous growth of the Lewis lung tumour in C57BL mice chronically exposed to fresh cigarette smoke was increased above that in age-matched control mice. When murine sarcoma virus (Harvey) induced tumour cells were introduced to the lungs of groups of BALB/c mice, only mice chronically exposed to fresh cigarette smoke died with tumour cells in the lungs. Tumour cell growth in mice during short term cigarette smoke exposure was indistinguishable from that in controls.**

CIGARETTE smoking has been associated with an increased incidence of neoplasia in the respiratory system and elsewhere in the body (Royal College of Physicians, 1971; U.S. Public Health Service, 1971). Studies of the influence of cigarette smoke inhalation on pulmonary tumourigenesis in mice exhibiting a relatively high natural incidence of lung adenomata and adenocarcinomata showed an earlier onset and increased incidence of these neoplasms (Leuchtenberger and Leuchtenberger, 1970). However, when a strain of mice with a low natural incidence of these neoplasms was examined, no increase in neoplasms was found (Leuchtenberger, Leuchtenberger and Rossier, 1973). Aqueous extracts of cigarette smoke condensate fed to rats have been found to increase the incidence of tumour production by chemical carcinogens (Sydnor, Allen and Higgens, 1972). The cigarette smoke product(s) could promote carcinogenesis by acting directly on malignant or premalignant cells, or by modifying some regulatory influence of the body opposing the growth of the neoplasm.

We now report the ability of transplantable tumours to grow in mice after short term or chronic cigarette smoke inhalation. The ability of tumours to grow subcutaneously and metastasize to the lungs, as well as the growth of cells introduced directly into the pulmonary system, have been examined.

### MATERIALS AND METHODS

*Cigarette smoke inhalation.*—Strictly line bred BALB/c and C57BL mice were exposed for 7–8 min on weekdays to fresh cigarette smoke in a Hamburg II small animal smoking machine set to give a 1 : 7, smoke : air ratio as previously described (Thomas, Holt and Keast, 1973*a*). The cigarette smoke exposures were continued throughout the duration of all experiments.

*Tumours and inoculation procedures.*—The Lewis lung tumour of C57BL mice was obtained from Dr Hellman, Imperial Cancer Research Fund, and maintained in our laboratories by serial passage through adult mice. This tumour originated as a spontaneous anaplastic lung carcinoma (Sugiura and Stock, 1955). Preliminary studies showed that the subcutaneous inoculation of $10^6$ viable cells could produce tumours in 18/20 mice. This number of cells was used as the standard inoculum in all experiments. The diameter of the tumours was determined by 2 measurements taken at rightangles to each other, employing calipers. The cube of the mean of the diameters determined by this method and the weight of the tumours were found to have a product moment correlation coefficient of 0·89.

The number of metastases in the lung was determined by the method of Wexler (1966); the lungs were infused with dilute India ink through the trachea, washed, fixed and bleached. The metastases appeared as white nodules on a black background. In order to allow the tumour maximum opportunity to metastasize, the tumour bearing animals were left until a number died or became moribund (about 30 days). All the mice in the test and control groups were then examined for lung secondaries and the significance of the difference between the number of metastases in the groups determined by Student's "*t*" test.

The TKL5 tumour cells were a clone from a line (W47-A) of tumour cells cultivated *in vitro* from a tumour induced in a BALB/c mouse by murine sarcoma virus (Harvey) (MSV-H) (Thomas *et al.*, 1973). They were maintained in tissue culture and produced virus. These tumour cells were introduced directly into the respiratory tract. Mice were anaesthetized with pentobarbitone sodium (Nembutal, Abbott Laboratories, Australia) and a blunt 19 gauge needle inserted under the epiglottis and almost to the bottom of the trachea where $10^5$ cells in 0·01 ml of media were introduced.

### RESULTS

C57BL mice were exposed to fresh cigarette smoke for 3 days, 23 weeks and 38 weeks before the subcutaneous inoculation of $10^6$ Lewis lung tumour cells. Cigarette smoke exposure was continued during tumour growth. The growth of the tumours in mice exposed to cigarette smoke for 3 days before the inoculation was almost identical with the growth of tumours in age-matched control mice (Fig.). However, the growth of the tumours in mice that had been exposed to fresh cigarette smoke for 23 and 38 weeks was significantly increased compared with control animals. The mice examined for metastases in the 2 smoke exposed groups had 2·8 ± 1·1 (mean ± s.e.) metastases per lung. The corresponding age-matched control mice had 0·8 ± 0·4 metastases per lung.

FIG.—The growth of Lewis lung tumour in mice exposed to cigarette smoke for 3 days, 23 weeks and 38 weeks. Each result is the mean ± s.e. of the tumour diameter of 10 mice: ● smoke exposed, ○ control. The significance of the differences between tumour diameter in the cigarette smoke exposed and control mice was determined by Student's "*t*" test. There was no significant difference between the diameter of the tumour in mice exposed to cigarette smoke for 3 days and the controls. The diameter of the tumours in mice exposed to cigarette smoke for 23 weeks and 38 weeks became significantly different ($P < 0 \cdot 05$) from the corresponding control mice on Days 20 and 9 respectively.

BALB/c mice were exposed to fresh cigarette smoke for 20 weeks before the introduction of $10^5$ TKL5 cells into the trachea. As shown in the Table (Expt 1), 6/13 of the smoke exposed mice died or became moribund. None of the age-matched control mice died within 50 days.

TABLE—*The Response of Cigarette Smoke Exposed Mice to an Intratracheal Inoculation of TKL5 Tumour Cells*

| | Smoke exposure | No. dead* / No. inoculated | Days to death Mean (range) |
|---|---|---|---|
| Expt 1 | 20 weeks | 6/13 | 28·6 (14–40) |
| | Control | 0/19 | |
| Expt 2 | 31 weeks | 6/15 | 15·0 (10–46) |
| | 3 days | 0/20 | |
| | Control | 0/20 | |

* Number of mice dead or moribund.

In another experiment (Table, Expt 2) a group of BALB/c mice were exposed to fresh cigarette smoke for 31 weeks before receiving the intratracheal inoculation of TKL5 cells. As well as an age-matched control group, this experiment included a group of mice of the same age as the control mice but exposed to cigarette smoke for 3 days before the inoculation. Only the mice exposed to fresh cigarette smoke for 31 weeks died or became moribund (6/15). The lungs of these mice were examined histologically. The lungs of all the mice examined exhibited numerous foci of neoplastic proliferation. These foci varied in size from a few cells to large extensive zones of neoplastic growth which completely replaced the whole lobes of the lung. Generally, however, they were small, consisting of 50–200 cells and were situated near bronchi or small vessels. The cells were spindly or irregular in shape, with large irregular nuclei and basophilic cytoplasm. Mitoses were present. In cases where foci were large, vasoproliferation became evident together with an infiltrate of lymphocytes, macrophages and giant cells. In addition, patches of bronchopneumonia and pulmonary collapse were commonly found, which probably contributed to the death of the animals.

### DISCUSSION

The results show that the subcutaneous growth of the transplantable Lewis lung tumour in mice was increased by chronic cigarette smoke inhalation. The mice chronically exposed to cigarette smoke had an increased number of metastases in the lung but this was not statistically significant. However, MSV-H tumour cells introduced directly to the lungs were able to persist and develop only in the lungs of mice chronically exposed to cigarette smoke. Other studies in our laboratories (unpublished) suggest that this is not caused by a decrease in tracheobronchial clearance. Short term cigarette smoke inhalation did not produce these effects. It has been shown that handling stress during cigarette smoke inhalation experiments does not affect body weight (Leuchtenberger and Leuchtenberger, 1970) or immune responses of mice (Esber *et al.*, 1973; Thomas *et al.*, 1974). This concurs with these results showing that chronic, rather than short term inhalation of cigarette smoke was required to enhance tumour growth.

While it is now well established that tobacco contains many carcinogenic substances (United States Public Health Service, 1971), the present experiments indicate that cigarette smoke inhalation was capable of modifying conditions within the smoking animal that result in promotion of growth of an established malignant cell. The fact that conditions for the production of enhanced tumour growth do not occur after short term smoke exposure (3 days) indicates that either an accumulation of substances acting directly on the tumour cells must occur, or that the cigarette smoke inhalation eventually impairs a mechanism controlling tumour growth. We have previously shown that the immune system of mice is impaired by chronic cigarette smoke inhalation (Thomas *et al.*, 1973*a*, *b*; 1974).

The immune system is thought to exert control on the growth of malignant cells (Burnet, 1970; Keast, 1970). As immunostimulants are known to inhibit the growth of the Lewis lung tumour (Renoux and Renoux, 1972), and the tumour cells produced by MSV are very antigenic (Fefer, McCoy and Glynn, 1967; Law, Ting and Stanton, 1968), it is possible that the cigarette smoke inhalation depressed the ability of the mice to elicit an immune response against the tumour cells. Not all types of neoplasia have been associated with cigarette smoking. However, the effect of immunosuppression on the expression of neoplasia would depend on the amount of control normally exerted by the immune system on that type of neoplasm and the amount of immune control required to prevent tumour growth.

This work was supported by the Australian Tobacco Research Foundation. WRT is in receipt of a C.S.I.R.O. postgraduate studentship.

### REFERENCES

BURNET, F. (1970) *Immunological Surveillance.* New York, London: Pergamon Press.

ESBER, H., MENNINGER, F., BOGDEN, A. & MASON, M. (1973) Immunological Deficiency Associated with Cigarette Smoke Inhalation of Mice. *Archs envir. Hlth*, **27**, 99.

FEFER, A., McCOY, J. & GLYNN, J. (1967) Induction and Regression of Primary Moloney Sarcoma-virus Induced Tumors in Mice. *Cancer Res.*, **27**, 1626.

KEAST, D. (1970) Immunosurveillance and Cancer. *Lancet, ii*, 710.

LAW, L., TING, R. & STANTON, M. (1968) Some Biologic, Immunogenic and Morphologic Effects in Mice after Infection with a Murine Sarcoma Virus 1. Biologic and Immunogenic Studies. *J. natn. Cancer Inst.*, **40**, 1101.

LEUCHTENBERGER, C. & LEUCHTENBERGER, R. (1970) Effects of Chronic Inhalation of Whole Fresh Cigarette Smoke and of its Gas Phase on Pulmonary Tumorigenesis in Snell's Mice. In *Morphology of Experimental Carcinogenesis.* Ed. P. Nettesheim, M. Hann and J. Deatherage. U.S. Atomic Energy Commission.

LEUCHTENBERGER, C., LEUCHTENBERGER, R. & ROSSIER, J. (1973) Enhancement of Pulmonary Carcinogenesis in Snell's Mice and its Absence in C57 Black Mice after Chronic Inhalation of Cigarette Smoke. *Proc. Am. Ass. Cancer Res.*, **14**, 6.

ROYAL COLLEGE OF PHYSICIANS (1971) *Smoking and Health Now.* London: Pitman Medical and Scientific Publishing Co.

RENOUX, G. & RENOUX, M. (1972) Levamisole Inhibits and Cures a Solid Malignant Tumor and its Pulmonary Metastases in Mice. *Nature, New Biol.*, **240**, 217.

SUGIURA, K. & STOCK, C. (1955) Studies on a Tumor Spectrum. III. The Effect of Phosphoramides on the Growth of a Variety of Mouse and Rat Tumors. *Cancer Res.*, **15**, 33.

SYDNOR, K., ALLEN, C. & HIGGENS, B. (1972) Effect of an Aqueous Extract of Cigarettes Smoke Condensate on Benzo[α]pyrene Induced Sarcoma and Body Weight in the Rat. *J. natn. Cancer Inst.*, **48**, 893.

THOMAS, W., AW, E., PAPADIMITRIOU, J. & SIMONS, P. (1973) *In vivo* and *in vitro* Studies on the Morphogenesis of Tumors Induced by Murine Sarcoma Virus (Harvey). *J. natn. Cancer Inst.*, **51**, 1541.

THOMAS, W., HOLT, P. & KEAST, D. (1973*a*) Effect of Cigarette Smoking on Primary and Secondary Humoral Responses of Mice. *Nature, Lond.*, **243**, 240.

THOMAS, W., HOLT, P. & KEAST, D. (1973*b*) Cellular Immunity in Mice Chronically Exposed to Fresh Cigarette Smoke. *Archs envir. Hlth*, **27**, 372.

THOMAS, W., HOLT, P. & KEAST, D. (1974) The Development of Alterations in the Primary Immune Response of Mice by Exposure to Fresh Cigarette Smoke. *Int. Archs Allergy appl. Immun.*, **46**, 481.

U.S. PUBLIC HEALTH SERVICE (1971) *The Health Consequences of Smoking.* Department of Health Education and Welfare Publication No. 71, p. 7513.

WEXLER, H. (1966) Accurate Identification of Experimental Pulmonary Metastases. *J. natn. Cancer Inst.*, **36**, 541.